# A Report from the Case Book of Thos. S. Powell, M. D., Sparta, Georgia

**Published:** 1855-09

**Authors:** 


ARTICLE III.—*A Report from the Case Book of* THOMAS S. POWELL, M. D., Sparta, Georgia.

MESSRS. EDITORS: Having become a subscriber to your publication, the "Atlanta Medical and Surgical Journal," and deeming it highly incumbent upon each subscriber to contribute his quota of means and matter towards promoting the object of your enterprise, I send you the names of four new subscribers, with a brief account of the first case that came under my treatment.

In the spring of 1846 I was summoned in great haste to visit my first patient, a wagon boy, who was reported to be dying at his camp, about a half mile from my office. On arriving, I was informed by the old wagoner that the boy was dead, and that he had just laid him out on a plank, in a yard belonging to an old free negro that lived a short distance from the camp, and that he would thank me to go and examine him, and write to his master that his death was not occasioned by any violence inflicted upon his person, but that he died from disease. To accommodate the old man, who seemed to be in great trouble, I rode down to the little hut, (which I imagined resembled very much those that the great and good Bœrhave, Fothergill, and Cullen, had been wont to enter,) and there I found the boy in the condition the old man had described—laid out on a plank—abdomen enormously swollen—cold, speechless, and pulseless—*dead* to all appearance.

Not finding any external wounds or bruises, I at once came to the conclusion, from the condition of the abdomen, that he was probably laboring under an attack of colic, and that the vital functions were held in suspension from its violence. I ordered him to be carried into the house, and a large tub of warm water to be immediately supplied. I then hurried back to my office, obtained a syringe, and returned in twenty minutes, finding my patient in the same condition. The water having been prepared, I had him placed in it at once, rubbed, and moved about actively for ten or fifteen minutes. He was then taken out and wrapped in blankets. I still, however, found no alteration in his condition. I then administered a clyster, composed of two table-spoonsful of Epsom Salts, dissolved in about three gills of corn-meal gruel, with the addition of a table spoonful of lard. To my great surprise, in about fifteen minutes, my patient opened his eyes, and made an effort to get up, but was so swollen and stiff that he could rise no higher than his elbow. I ordered the attendants to raise him to his feet, and he then immediately pointed out of the window. I told them to carry him out of the house. Being unable, from tension and tenderness, to sit down, they held up while he discharged from the bowels an immense amount of fecal matter and half digested fruit. He was then able to speak, and his pulse could be felt. He was carried back, put to bed, and warm fomentations applied to his bowels.

In about four hours I called again, and found him very comfortable; with very little pain; pulse good; slightly accelertaed; abdomen soft; no thirst; tongue moist and covered with a creamy coat. I ordered a table-spoonful of Castor Oil with ten or fifteen drops of Laudanum.

I saw him again on the next morning and found him still improving. The medicine had acted twice during the night; his respiration was free and natural; strength such as to enable him to get in and out of bed without assistance. He desired food, and was allowed a cup of coffee and a few batter cakes. I dismissed him, and he left for home in a few hours.

Messrs. Editors, I have not selected this case for the first number of your Journal because it would furnish any light upon the treatment of colic, but to remind some of your young readers of the old adage, "that there is hope as long as life remains," though it *may be* almost imperceptible, if the proper means are applied with energy and perseverance.

I could give you many cases that have occurred in my practice in Hancock, that have recovered after being reported as dying, whose recovery I can attribute to nothing but the lesson of perseverance I learned in this my first case.

Perseverance is essential to the success of any enterprise, however popular it may be, or talented and gifted its projectors. No doubt you will meet with many difficulties in your noble and spirited enterprise. I know Colleges and Medical Journals in the South are rather below par. Editors and professors complain sadly of a want of patronage.

There seems a fatality attending such undertakings in our latitude, but not the less bold should you be for all that. "Old fogies" will cry "you will fail," but with perseverance *you must*, you will, succeed in establishing both your College and Journal upon a sure and permanent foundation, and be able to sneer at the word "FAIL," and point proudly to the students in your College Hall and the long list of names upon your subscription book as an evidence of your energy and perseverance.

